# Transcriptome analysis reveals molecular mechanisms of lymphocystis formation caused by lymphocystis disease virus infection in flounder (*Paralichthys olivaceus*)

**DOI:** 10.3389/fimmu.2023.1268851

**Published:** 2023-10-05

**Authors:** Honghua Zhang, Xiuzhen Sheng, Xiaoqian Tang, Jing Xing, Heng Chi, Wenbin Zhan

**Affiliations:** ^1^ Laboratory of Pathology and Immunology of Aquatic Animals, Key Laboratory of Mariculture, Ministry of Education (KLMME), Ocean University of China, Qingdao, China; ^2^ Function Laboratory for Marine Fisheries Science and Food Production Processes, Qingdao National Laboratory for Marine Science and Technology, Qingdao, China

**Keywords:** flounder (*Paralichthys olivaceus*), lymphocystis disease virus, RNA-Seq, transcriptome, lymphocystis formation, molecular mechanism

## Abstract

Lymphocystis disease is frequently prevalent and transmissible in various teleost species worldwide due to lymphocystis disease virus (LCDV) infection, causing unsightly growths of benign lymphocystis nodules in fish and resulting in huge economic losses to aquaculture industry. However, the molecular mechanism of lymphocystis formation is unclear. In this study, LCDV was firstly detected in naturally infected flounder (*Paralichthys olivaceus*) by PCR, histopathological, and immunological techniques. To further understand lymphocystis formation, transcriptome sequencing of skin nodule tissue was performed by using healthy flounder skin as a control. In total, RNA-seq produced 99.36%-99.71% clean reads of raw reads, of which 91.11%-92.89% reads were successfully matched to the flounder genome. The transcriptome data showed good reproducibility between samples, with 3781 up-regulated and 2280 down-regulated differentially expressed genes. GSEA analysis revealed activation of Wnt signaling pathway, Hedgehog signaling pathway, Cell cycle, and Basal cell carcinoma associated with nodule formation. These pathways were analyzed to interact with multiple viral infection and tumor formation pathways. Heat map and protein interaction analysis revealed that these pathways regulated the expression of cell cycle-related genes such as *ccnd1* and *ccnd2* through key genes including *ctnnb1*, *lef1*, *tcf3*, *gli2*, and *gli3* to promote cell proliferation. Additionally, cGMP-PKG signaling pathway, Calcium signaling pathway, ECM-receptor interaction, and Cytokine-cytokine receptor interaction associated with nodule formation were significantly down-regulated. Among these pathways, *tnfsf12*, *tnfrsf1a*, and *tnfrsf19*, associated with pro-apoptosis, and *vdac2*, which promotes viral replication by inhibiting apoptosis, were significantly up-regulated. Visual analysis revealed significant down-regulation of *cytc*, which expresses the pro-apoptotic protein cytochrome C, as well as *phb* and *phb2*, which have anti-tumor activity, however, *casp3* was significantly up-regulated. Moreover, *bcl9*, *bcl11a*, and *bcl-xl*, which promote cell proliferation and inhibit apoptosis, were significantly upregulated, as were *fgfr1*, *fgfr2*, and *fgfr3*, which are related to tumor formation. Furthermore, RNA-seq data were validated by qRT-PCR, and LCDV copy numbers and expression patterns of focused genes in various tissues were also investigated. These results clarified the pathways and differentially expressed genes associated with lymphocystis nodule development caused by LCDV infection in flounder for the first time, providing a new breakthrough in molecular mechanisms of lymphocystis formation in fish.

## 1 Introduction

Lymphocystis disease virus (LCDV) is a member of the genus *lymphocystivirus* of the *Iridoviridae* family and the causative agent of lymphocystis disease (1). LCDV has infected more than 140 species of marine and freshwater fish worldwide, including flounder (*Paralichthys olivaceus*), which is economically important in Asian countries such as Japan, Korea, and China, causing great economic losses (2, 3). Fish with lymphocystis disease develop benign cauliflower-like nodules on the skin, gills, fins, mouth, and some internal organs (4). LCDV mainly infects fibroblasts, and a large number of fibroblasts exist in the connective tissue of the skin, so the skin is the main target tissue where the presence of lymphocystis nodules is usually observed (4). These nodules vary in size, present creamy white or pink (vascularly congested), and are either dispersed or aggregated into clusters. Histopathologically, the nodules contain a large number of hypertrophied cells (i.e., lymphocystis cells) developed from the infected fibroblasts in the connective tissue underlying the epithelium, which may be up to 100 times the size of normal cells in fish, with a thick hyaline capsule outside the cell membrane, expanded and irregular nuclei, and cytoplasm containing a large number of viral particles and inclusion bodies (5–8). Although lymphocystis disease is a self-limiting and rarely fatal disease, and the lesions can heal and the fish may recover after a few weeks, the benign nodules lead to nonmarketability of diseased fish, poor growth, and secondary bacterial infections that can lead to mortality (2, 3). To elucidate the mechanism of LCDV infection and develop effective preventive measures, considerable research has focused on LCDV-host interaction. Remarkably, a 27.8 kDa protein on the cell membrane of flounder gill (FG) cells is identified as the cellular receptor for LCDV entry, which is further confirmed to be voltage-dependent anion channel protein 2 (VDAC2) and receptor of activated protein C kinase 1 (RACK1), and the two receptors mediate LCDV invasion of host cells through interacting with a 32kDa viral adhesion protein (VAP) of LCDV that encoded by LCDV ORF038 gene, using the caveolae/raft mediated endocytosis pathway into FG cells (9–12). In addition to fibroblasts, which are considered to be the main target cells of LCDV, some studies have shown that gilthead seabream (*Sparus aurata*) hepatocytes, macrophages, and *in vitro*-cultured leukocytes are involved in LCDV infection (13, 14). Recently, LCDV has been found to infect peripheral blood IgM^+^ B cells of flounder, and IgM^+^ B cells also express the 27.8 kDa receptor protein and support LCDV replication, so B cells may be the vector of LCDV transmission among tissues (15). However, the process by which LCDV infects host cells and proliferates intracellularly resulting in hypertrophied cells and then the formation of lymphocytsis nodule, and the underlying mechanism regulating lymphocytsis formation, remains to be clarified.

In mammals, viral infections are a major contributor to nodules and even malignant tumors. Studies have shown that the known viruses, e.g., Epstein–Barr virus (EBV), Merkel cell polyomavirus (MCPyV), hepatitis B virus (HBV), hepatitis C virus (HCV), human T-lymphotropic virus 1 (HTLV-1), human papillomaviruses (HPVs) and Kaposi sarcoma-associated herpesvirus (KSHV), can promote tumorigenesis through common host cellular targets and pathways (16). These viruses have been found to support their proliferation by controlling the cell cycle, apoptosis, autophagy, DNA damage, immune escape (viral protein homologs that regulate immune mechanisms), and biosynthesis and metabolism (17). In fish, few viruses cause nodules but LCDV, so there are fewer studies on the mechanisms of lymphocystis nodule formation. Previously, microarray experiments were used to track the formation of lymphocystis cells, and it was concluded that apoptosis and division were inhibited in the ventral fins of LCDV-infected flounder and further formed lymphocystis cells by cell fusion (5), but the mechanism of nodule formation remains to be systematically investigated.

RNA-Seq is a technique for the detection of transcriptome expression levels in samples by high-throughput sequencing (18) and has become a revolutionary tool for transcriptional and genomic characterization (19, 20). This technique is very sensitive and can accurately detect rare transcripts, with a very wide range of detection and high sensitivity to gene expression at high or very low levels (18). Using RNA-Seq techniques, we have studied gill tissue differentially expressed genes (DEGs) in flounder at one week post LCDV infection, providing preliminary insights into fish defense mechanisms against LCDV (6).

In this study, the flounder (*P. olivaceus*) naturally developed skin and fin nodules for about one month in a fish farm in Rizhao, Shandong province of China. The flounder was first detected for LCDV infection by using polymerase chain reaction (PCR), quantitative real-time PCR (qRT-PCR), histopathological and indirect immunofluorescence assay (IFA) technique. Subsequently, transcriptomic data of skin nodule tissues of LCDV-infected flounder were obtained by high-throughput sequencing with the skin of healthy flounder as control, the pathways and differentially expressed genes associated with nodule formation were analyzed, and expression patterns of focused genes in various tissues were also analyzed.

## 2 Materials and methods

### 2.1 Experimental fish and sampling

Healthy and diseased flounder (250 ± 50 g) were taken from the fish farm, and cultured in a continuous aerated and flow-through seawater system at a temperature of 21 ± 1°C. Before all, flounders were tested by PCR to confirm LCDV-free in the healthy fish and LCDV infection in the diseased fish. For RNA-Seq, skin nodule tissues from four diseased fish and skin tissues from four healthy fish, named LS (LCDV-infected) and CS (control) groups respectively, were randomly selected and placed in liquid nitrogen for rapid freezing. For qRT-PCR, flounder were anesthetized with 100 mg/mL MS-222 (Sigma, MO, USA), and the liver, spleen, head kidney, trunk kidney, hindgut, gills, and skin were taken from these fish. The samples were rapidly immersed in RNAlater (Thermo Scientific, Waltham, Massachusetts, USA) and stored at -80°C.

### 2.2 Histological preparation and indirect immunofluorescence assay

Skin nodule tissue from diseased fish was aseptically excised, washed with phosphate-buffered saline (PBS), fixed in Bouin’s solution, and then washed several times with 70% ethanol. The tissue was subsequently dehydrated in a series of increasing concentrations of ethanol, cleared in xylene, and embedded in paraffin by conventional procedures. The 5 μm-thick sections were cut, and stained with hematoxylin-eosin (H-E) and histologically observed by Zeiss microscope (Oberkochen, Germany).

For IFA, at least three paraffin sections of each fish were subjected to IFA using mouse anti-LCDV 32kDa VAP monoclonal antibody (Mab) 1C8 previously prepared in our laboratory (21). Briefly, the sections were dewaxed using xylene and rehydrated in decreasing concentrations of ethanol, finally washed in PBS. The antigen repair of the sections was carried out by using modified sodium citrate antigen repair solution (50×) (1:50, Beyotime, Shanghai, China) at 95°C for 20 min, followed by incubation with 4% bovine serum albumin (BSA) in PBS at 37°C for 1 h. Mouse anti-LCDV 32kDa VAP Mab 1C8 (1:200 diluted in PBS) was incubated as primary antibody at 37°C for 1 h. After three washes with PBST (PBS containing 0.05% Tween-20) for 5 min each, FITC-conjugated goat-anti-mouse Ig (1:1000 diluted in PBS, Sigma, MO, USA) was incubated as secondary antibody for 45 min at 37°C. Subsequently, DAPI (1:1000 diluted in PBS, Thermo Scientific, Waltham, Massachusetts, USA) was stained for 15 min at room temperature in the dark to visualize the cell nucleus. Non-immune mouse serum replacing primary antibody was used as a negative control. Finally, slides were mounted with extended glass mounting medium (Thermo Scientific, Waltham, Massachusetts, USA) and fluorescence imaging was performed under an immunofluorescence microscope (Zeiss, Oberkochen, Germany).

### 2.3 Transmission electron microscopy

Skin lymphocystis nodule tissue less than 1 mm^3^ in volume was excised, rinsed with PBS to remove blood and mucus, and then fixed with 2.5% glutaraldehyde in 0.1 mol/L PBS (pH 7.4) for 2 h and post-fixed with 1% osmium acid in PBS at 4°C. The samples were dehydrated in gradient alcohol, embedded in Epon812 embedding agent, and sectioned on an ultrathin microtome. Finally, the ultra-thin sections were stained with uranyl acetate-lead citrate and observed by transmission electron microscope.

### 2.4 Genetic level detection of LCDV

Total DNA was extracted from the skin nodule tissues of LS groups and the skin of CS groups. LCDV ORF038 gene encoding the LCDV 32 kDa VAP was used for PCR amplification (F: 5’-ATGTCTGTCATAGGATTTACTCTACAA-3’, R: 5’-AAAAGTCAAATAAAATATTAAAATCATT-3’). 20 μL PCR reaction system: DNA template 1 µL, each primer 1 µL, Ex Taq DNA polymerase 0.5 µL (TaKaRa, Japan), Ex Taq buffer 2.5 µL, dNTPs 2 µL, ddH_2_O 12 μL. Reaction procedure: 95°C for 5 min, 35 amplification cycles (95°C for 30 sec, 60°C for 30 sec, 72°C for 1 min), 72°C for 10 min. The PCR product size was detected by 1.0% agarose gel electrophoresis.

To determine the replication of LCDV in various tissues of the diseased flounder, total DNA was extracted from liver, spleen, head kidney, trunk kidney, hindgut, gill and skin tissues, followed by 50 ng DNA as template with a pair of specific primers (F: 5’- TCTTGTTCAGCATTTACTTCTCGGC -3 ‘ and R: 5’- TCTTCTCCTTTAGATGATTTCCC -3’) (11) for qPCR amplification of the LCDV ORF038 gene fragment. Each sample was tested four times, and non-infected samples served as negative controls. After amplification, a melting curve analysis was undertaken to guarantee that there was no non-specific amplification. Finally, the LCDV copy number was determined by calculating the Ct value from the previously established standard curve (11). The data was expressed as mean log_10_ copies/50 ng DNA.

### 2.5 cDNA library construction and sequencing

Total RNA was isolated from flounder skin nodule tissue according to the manufacturer’s protocol using the Trizol kit (Invitrogen, Carlsbad, CA, USA). The quality of the RNA was determined using an Agilent 2100 Bioanalyzer (Agilent Technologies, Palo Alto, CA, USA) and RNase-free agarose gel electrophoresis. Afterward, the mRNA was enriched by magnetic beads with Oligo(dT) and then interrupted with First Strand Synthesis Reaction Buffer. To synthesize the first strand of cDNA in the M-MuLV reverse transcriptase system, the fragmented mRNA was used as a template, and random oligonucleotides were used as primers. The RNA strand was then degraded with RnaseH, and the cDNA second strand was synthesized with dNTPs under the DNA polymerase I system. Sequencing connectors were ligated and poly(A) was added. The purified double-stranded cDNA was end-repaired. Approximately 200 bp cDNAs were screened with AMPure XP beads, amplified by PCR, and then sequenced using Illumina HiSeq2500 in Gene Denovo Biotechnology Co (Guangzhou, China).

### 2.6 Filtering and alignment of clean reads

Reads obtained from the sequencing machines include raw reads containing adapters or low-quality bases. These raw reads will affect the following assembly and analysis. Reads were therefore further filtered using fastp (version 0.18.0) to obtain high-quality clean reads (22). The criteria were as follows: eliminating adapter-containing reads, reads with more than 10% of unknown nucleotides (N), and reads with more than 50% of low-quality (Q-value ≤ 20) bases.

The ribosomal RNA (rRNA) database was mapped using the short reads alignment program Bowtie2 (version 2.2.8) (23) before the rRNA-mapped reads were eliminated. The remaining clean reads were then used for gene abundance estimation and assembly. Using HISAT (version 2.2.4) with “-rna-strandness RF” and other default options, paired-end clean reads were mapped to the reference genome after creating an index of the reference genome (24).

### 2.7 Differentially expressed genes and gene set enrichment analysis

Differential gene expression between the CS and LS groups was analyzed using DESeq2 software (25). Genes with false discovery rate (FDR) < 0.05 and absolute fold change (FC) ≥ 2 were differentially expressed genes (DEGs). To determine whether a collection of genes in particular KEGG pathways had significant differences in the two groups, gene set enrichment analysis (GSEA) was carried out using the tools GSEA and MSigDB (26). In a nutshell, the SinaltoNoise normalization method was used to input the gene expression matrix and rank genes, and enrichment scores and p-value were calculated in default parameters.

### 2.8 Protein-protein interaction and pathway visualization

A protein-protein interaction (PPI) network was identified using String (version 10) (27), which determined genes as nodes and interactions as lines in a network. A core and hub gene biological interaction was shown using Cytoscape (version 3.7.1) software by visualizing the network file (28). Pathway visualization and data integration of genes based on gene expression using PATHVIEW (https://pathview.uncc.edu/, accessed on 30 May 2023) (29, 30).

### 2.9 Quantitative real-time PCR

Briefly, 1 μg of total RNA was extracted from liver, spleen, head kidney, trunk kidney, hindgut, gill, and skin tissues and reverse transcribed into cDNA in a 20 μL reaction system. 2 μL of cDNA was then used as a template. Primer Premier 5 was used to design specific primers for the pathway-related genes in focus. Expression levels were normalized using flounder *β-actin* as an internal reference. The qRT-PCR was performed using SYBR green Master Mix (Roche, Switzerland) in a LightCycler^®^ 480 II Real Time System (Roche, Switzerland). The 2^−△△Ct^ method was used to analyze the expression levels of the selected genes. The primers used in this section are shown in **Table 1**. Each sample was run in quadruplicate.

**Table 1:** Sequence information for primers used in this study.

Gene	Accession No.	Primer Sequence (5’->3’)	Length (bp)
β-actin	XM_020109620.1	F: GTCCCTGTATGCCTCTGGTC	215
		R: TGTCACGCACGATTTCCCTC	
wnt5a	XM_020096312.1	F: ACTTCCGCAAGGTGGGTGAT	178
		R: GAGCCCGTGCTTTGGTTCTT	
ctnnb1	XM_020090668.1	F: AGAAACGTCTGTCGGTGGA	161
		R: GCCGTAGGCTGATGGGTAT	
lef1	XM_020101320.1	F: ACTAAACGCCTTCATGCTC	160
		R: TTCCTTGCGGGCTAACT	
tcf3	XM_020107767.1	F: CCTGGATTTCAGTGCGATGT	109
		R: GCCGCTACGCTCGTCTATT	
gli2	XM_020109184.1	F: TTTACGAGACCAACTGCCACTG	147
		R: GCTTCTGCTCCCGAGAACACT	
ccnd1	XM_020080206.1	F: TACTGTGCTGCGAGGTGGACT	116
		R: GTTGGGAGACGGTAGGTAGGTTT	
ccnd2	XM_020078448.1	F: CATGTTCCTCGCATCCAAAT	114
		R: ACCACCAGTTCCCATTCCAG	
bcl9	XM_020089951.1	F: GGAATCCTGTTCCTGGCTCAA	181
		R: GCCTCCGTCTTCGGTTTAG	
bcl11a	XM_020088700.1	F: ACCACCCGAGTGCCTTTGA	182
		R: TGTCTGGAATGGCTGGAGTAA	
bcl-xl	XM_020111495.1	F: TGGTGGAGTTCTTTATCAGTTACA	133
		R: TTGACCAGCAAGCCATTACT	
fgfr1a	XM_020107117.1	F: CTGAAGGAAGGTCACCGTATG	194
		R: GAGTATTGGTCCAGAGGCAC	
fgfr3	XM_020108467.1	F: TCCTATGGTGTGTTGTTGTGG	138
		R: GTACAGCTCATGTGTGCAGTTTG	
fzd2	XM_020113633.1	F: CGATATGCTCAGCGGCGT	108
		R: AGGAACGAGGTCCCGATGAA	
fzd8	XM_020113464.1	F: ACAACTGCTCCAACTGTTTACTG	138
		R: ACGAGTCCAGGGTCTTGCC	
pik3r1	XM_020082241.1	F: TAGAGCCCTTGCCGAGAT	136
		R: TGGAGCAGCCTGACTTTCAC	
fgfr2	XM_020088824.1	F: GATCCACGCTGGGAGTTT	128
		R: TCACGGCTTCCTTAGGTTT	
mtor	XM_020088845.1	F: CAGCGTCAGAACAACCAAGCA	169
		R: AGCAGTGAAGGTGTCCCCA	
fzd9	XM_020095313.1	F: CGAAGCCCACAGCAACTAC	136
		R: CCCAGAGTCCATACTCCCTAC	
lrp5	XM_020104881.1	F: CATTGACTATGTAGACCATCGAC	150
		R: CAGTAGATAAAATCTTGGTATTGTG	
tcf7l2	XM_020083352.1	F: ACGAGACAACTATGCGGCTAAC	167
		R: GCCCGAACAAGGCACGAC	
mdm4	XM_020086872.1	F: ACAGAGGCAGCACATAGTCCA	140
		R: CAGCGTCAGAACAACCAAGCA	
ruvbl1	XM_020086807.1	F: AGGTGCCCTTCTGTCCTAT	117
		R: CCTTGGTTTCTTTGATACGC	

### 2.10 Statistical analysis

Statistical Products and Services Solutions (SPSS) software (version 20.0, IBM, BY, USA) was used to analyze the obtained data. One-way analysis of variance (ANOVA) was used to analyze the results of viral proliferation, and the expression levels of genes in each tissue. Values were deemed significant at *p* < 0.05.

## 3 Results

### 3.1 Infection characteristics of LCDV in flounder

**Figure 1:**
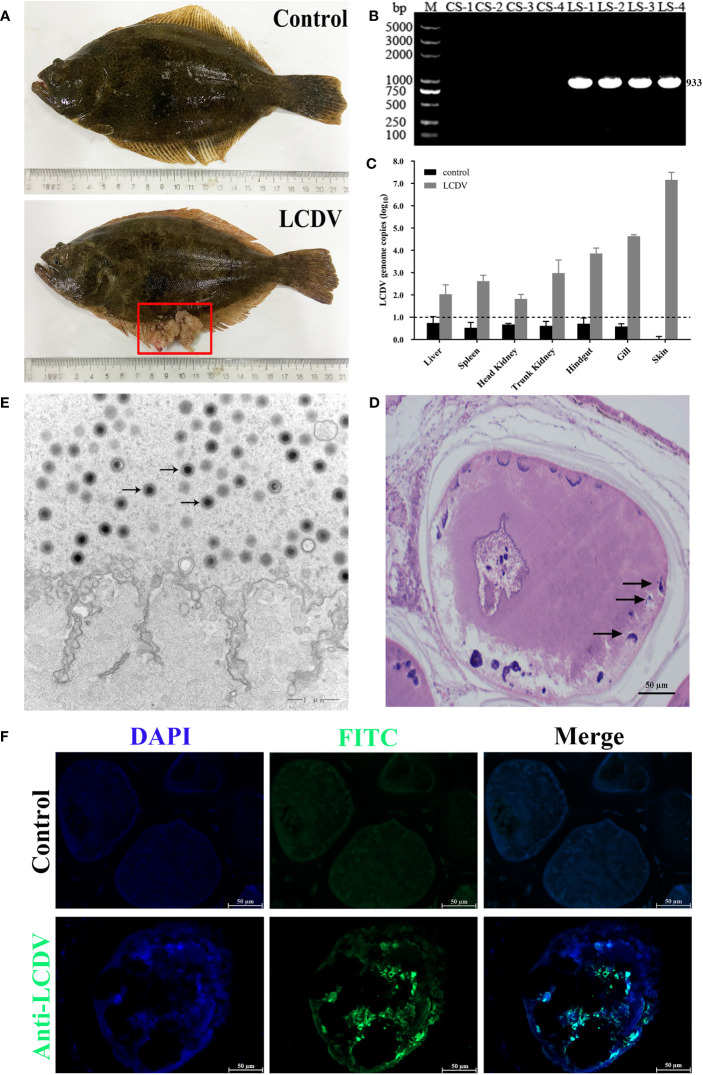
LCDV detection in flounder. **(A)** Clinical symptoms of naturally LCDV-infected flounder, and the healthy fish as control. The diseased fish showed obvious nodules on skin and fin (red box). **(B)** PCR assay of the skin of four healthy and diseased flounder, respectively. 933bp LCDV specific band was amplified in all LCDV-infected (LS) groups but not in control (CS) groups. M: DL5000 DNA Marker. **(C)** Copy numbers of LCDV in different tissues of the diseased flounder and control fish. Mean viral copy numbers were represented in the log_10_ scale. **(D)** H-E staining of skin nodule tissue of diseased fish. Basophilic inclusion bodies (black arrows) were present in the hypertrophic lymphocystis cells. Scale bar = 50µm. **(E)** Transmission electron microscope observation of skin nodule tissue. A large number of viral particles appeared in the cytoplasm (black arrows) of lymphocystis cells. Scale bar = 1µm. **(F)** skin nodule tissue fluorescently stained with mouse anti-LCDV 32kDa VAP Mab. Positive green signal was observed in the cytoplasm; non-immune mouse serum as negative control showing no green signal; cell nucleus stained with DAPI (blue); Scale bar = 50μm.

The healthy and naturally diseased flounder were tested for LCDV. Cauliflower-like nodules were evident on the skin and fins of the diseased flounder (**Figure 1A**), and a specific LCDV ORF038 gene band of 933 bp was amplified from the diseased fish but not in the healthy fish skin (**Figure 1B**). The lymphocystis cells within skin connective tissue had hypertrophic features with basophilic inclusion bodies in the cytoplasm by H-E staining (**Figure 1D**), and a large number of viral particles were observed in the cytoplasm by transmission electron microscopy (**Figure 1E**). Moreover, the cytoplasm of lymphocystis cells was positive for LCDV as shown by IFA (**Figure 1F**). The qRT-PCR results indicated that LCDV copy number was detected in all tested tissues of the affected fish, with the highest copy number of 3.0×10^7^/50 ng DNA in the skin, followed by the gills (4.5×10^4^), trunk kidney (1.5×10^4^), hindgut (1.1×10^4^), spleen (6.7×10^2^) and liver (3.0×10^2^), and the lowest in the head kidney (9.0×10^1^), while the controls had no LCDV proliferation (**Figure 1C**). All these results confirmed that the healthy fish were not infected by LCDV, while the diseased fish were naturally infected with LCDV, so they could be used for the next experiment.

### 3.2 Transcriptome sequencing quality

Clean reads with 40994192, 46893012, 40908092, and 49650708 were obtained from the CS group, and 52683364, 42481640, 47979600, and 39471322 were obtained from the LS group, respectively. Sequence data for CS-1, CS-2, CS-3, CS-4, LS-1, LS-2, LS-3, and LS-4 have been submitted to the NCBI database under accession numbers SAMN36345915, SAMN36345916, SAMN36345917, SAMN36345918, SAMN36345919, SAMN36345920, SAMN36345921, and SAMN36345922, respectively. The (guanine and cytosine) GC percentages for all samples ranged from 47.52%-49.46%, with reads over 96.0% for Q20 and 91.0% for Q30 for each sample, indicating high sequencing quality. Also, 91.11%-92.89% of the sequence reads were successfully localized to the flounder genome (**Table 2**).

**Table 2:** Summary of sequencing and assembly statistics for the transcriptome data.

Samples	Raw reads	Clean reads (%)	Q20 (%)	Q30 (%)	GC (%)	Total mapped (%)
CS-1	41131938	40994192 (99.67%)	97.90%	94.08%	47.93%	37936874 (92.84%)
CS-2	47039530	46893012 (99.69%)	97.94%	94.17%	49.08%	43447661 (92.86%)
CS-3	41052022	40908092 (99.65%)	97.91%	94.09%	48.85%	37935490 (92.89%)
CS-4	49796204	49650708 (99.71%)	96.92%	91.48%	48.33%	45105990 (91.25%)
LS-1	52959048	52683364 (99.48%)	97.34%	92.83%	49.46%	48216058 (91.85%)
LS-2	42753138	42481640 (99.36%)	97.15%	92.35%	47.52%	38516818 (91.11%)
LS-3	48225006	47979600 (99.49%)	97.35%	92.83%	48.58%	43851911 (91.71%)
LS-4	39663740	39471322 (99.51%)	97.36%	92.83%	49.00%	36186336 (92.00%)

### 3.3 Differentially expressed genes after LCDV infection

**Figure 2:**
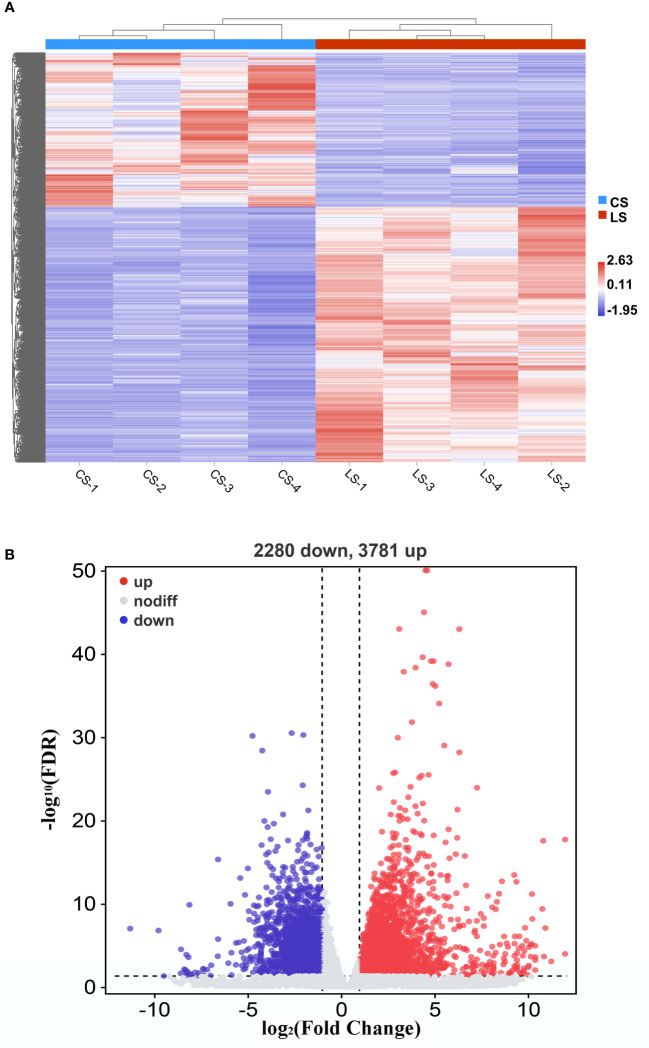
DEGs in flounder after LCDV infection. **(A)** The heat map represented the expression levels of those genes that were differentially expressed, as well as the normalization of gene expression using the z-score to construct hierarchical clusters of different samples. Two highly differential clusters were observed: one for genes that were inhibited following LCDV infection and the other for genes that were overexpressed following infection. **(B)** Volcano map of differentially expressed genes. Red represented up-regulated genes, blue represented down-regulated genes, and grey represented no differences.

When we analyzed the expression of different genes in LCDV-infected flounder versus healthy fish, a total of 6061 DEGs were observed. Hierarchical clustering of differential gene expression patterns was performed, and a heat map was used to present the clustering results. The heat map results showed that four biological replicates were clustered together in each group, indicating good concordance (**Figure 2A**). The volcano map results demonstrated that 3781 DEGs were significantly up-regulated and 2280 DEGs were significantly down-regulated after LCDV infection (FDR < 0.05, FC ≥ 2) (**Figure 2B**).

**Figure 3:**
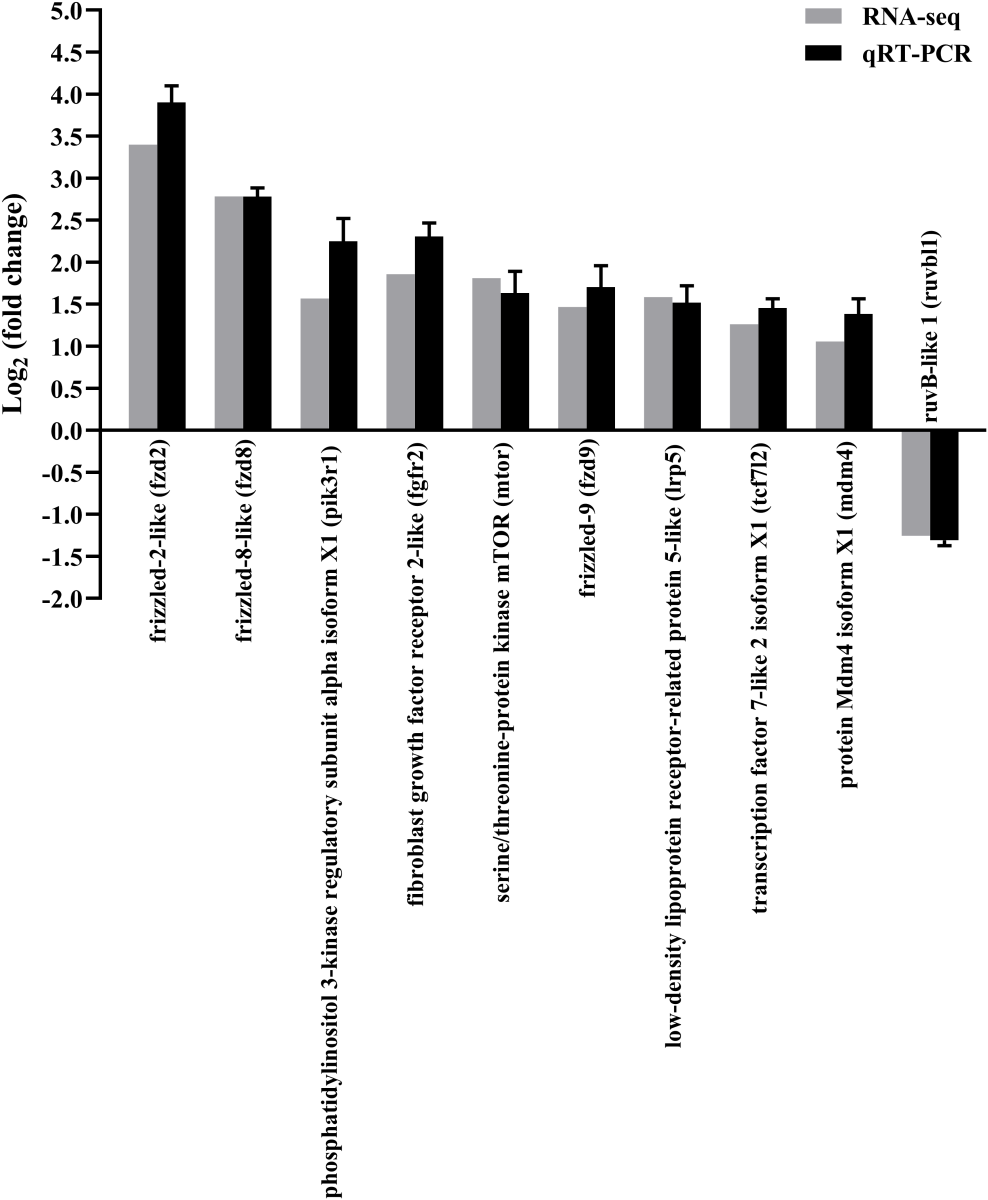
Confirmation of RNA-seq data by qRT-PCR. The results were presented as the means ± SEM of four individuals.

The qRT-PCR of random 10 DEGs validated these RNA-Seq results. The qRT-PCR results for the tested genes showed similar expression patterns as observed in the RNA-Seq data (**Figure 3**), indicating that the RNA-seq results were reliable.

### 3.4 Lymphocystis nodule-related activated pathways and genes in response to LCDV infection

**Figure 4:**
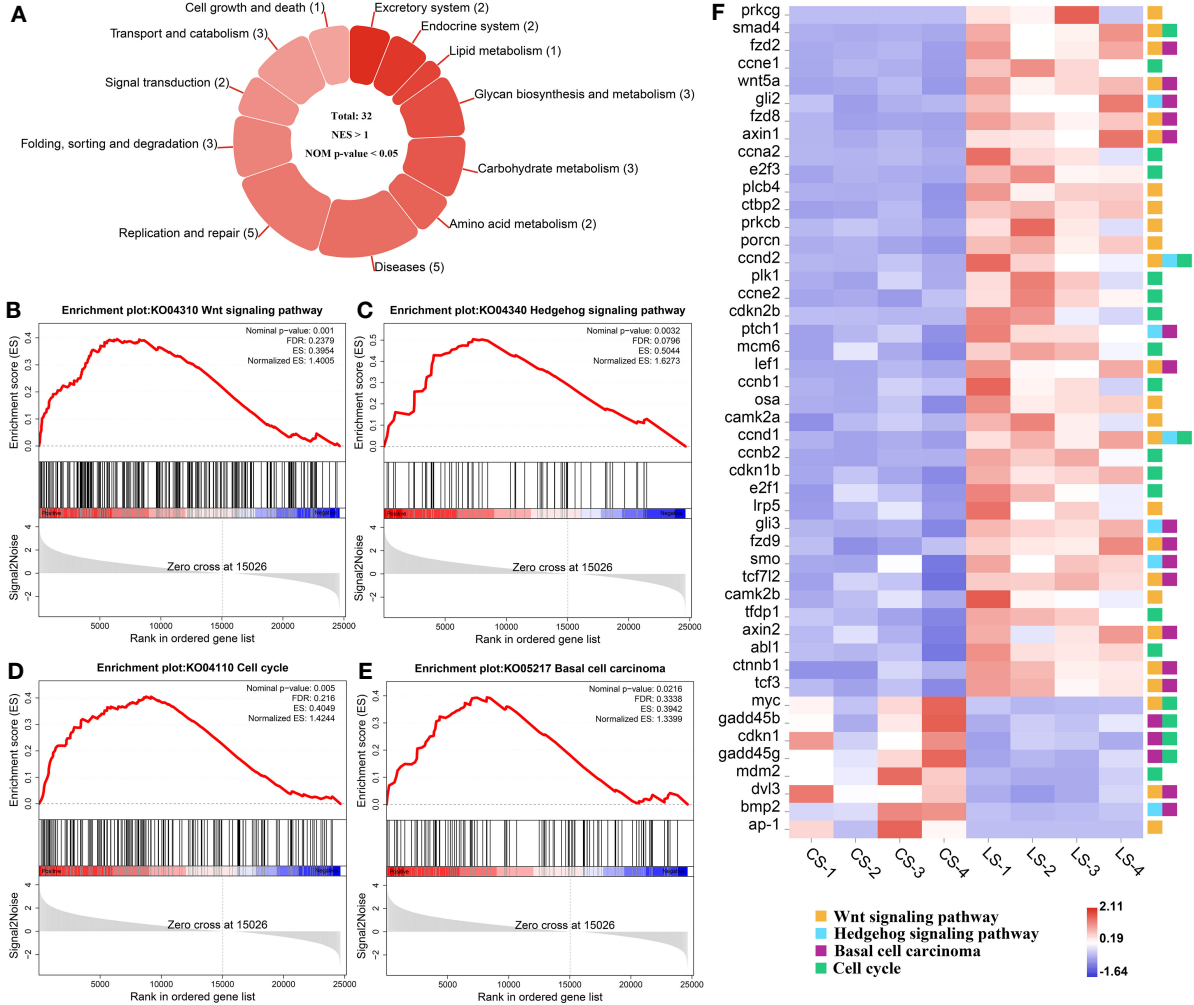
GSEA analysis of activated pathways and related genes that are associated with lymphocystis nodule formation after LCDV infection of flounder. **(A)** The number of activated pathways. **(B-E)** KEGG pathways associated with nodule formation including Wnt signaling pathway **(B)**, Hedgehog signaling pathway **(C)**, Cell cycle **(D)**, and Basal cell carcinoma **(E)**. **(F)** The heatmap of DEGs. GSEA was used to analyze the signaling pathway enrichment in different groups. Normalized enrichment score (NES) indicated the analysis results across gene sets. Nominal p-value presented if a set was significantly enriched.

The KEGG pathways that hosted the gene set of flounder after LCDV infection were analyzed using the GSEA method. In total, 32 active signaling pathways were found (**Figure 4A**). Among them, those related to nodule formation mainly included Wnt signaling pathway (**Figure 4B**) and Hedgehog signaling pathway (**Figure 4C**) in Signal transduction, Cell cycle (**Figure 4D**) in Cell growth and death, and Basal cell carcinoma (**Figure 4E**) in Diseases. In the four pathways, *prkcg*, *smad4*, *fzd2*, *ccne1*, *wnt5a*, *gli2*, *fzd8*, *axin1*, *ccna2*, *e2f3*, *plcb4*, *ctbp2*, *prkcb*, *porcn*, *ccnd2*, *plk1*, *ccne2*, *cdkn2b*, *ptch1*, *mcm6*, *lef1*, *ccnb1*, *osa*, *camk2a*, *ccnd1*, *ccnb2*, *cdkn1b*, *e2f1*, *lrp5*, *gli3*, *fzd9*, *smo*, *tcf7l2*, *camk2b*, *tfdp1*, *axin2*, *abl1*, *ctnnb1*, and *tcf3* had significantly upregulated levels of gene expression as compared with healthy groups, while *myc*, *gadd45b*, *cdkn1*, *gadd45g*, *mdm2*, *dvl3*, *bmp2*, and *ap-1* were significantly downregulated, and there were some shared DEGs between these four pathways (FDR < 0.05, FC ≥ 2) (**Figure 4F**). Details of these genes are listed in **Table S1**.

### 3.5 Analysis of protein and KEGG pathway interaction networks

**Figure 5:**
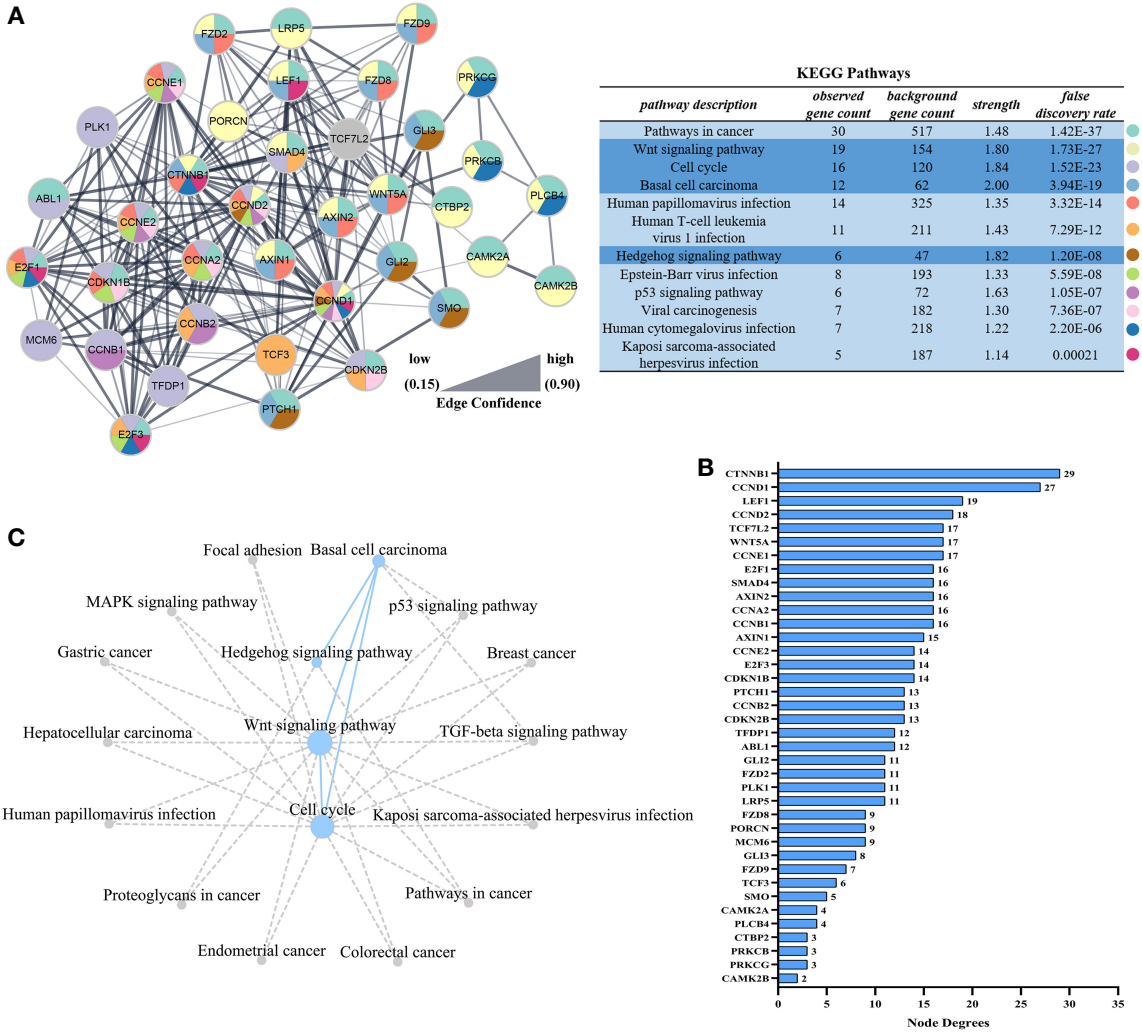
Interaction networks based on STRING analysis after LCDV infection of flounder. **(A)** Protein interaction network of up-regulated genes in **Figure 4F**. The table showed the KEGG analysis of the protein interaction network, dark blue was the pathway analyzed in **Figures 4B–E**. **(B)** Node degrees of protein interaction network. **(C)** The KEGG database was used to construct the pathway network in **Figures 4B–E**. The dotted line represented the presence of a relationship and the dot size represented the degree of connectivity.

In order to identify the key genes, PPI analysis of the appeal genes showed that *ctnnb1*, *ccnd1*, *lef1*, *ccnd2*, and *wnt5a* played key roles as hub genes (**Figures 5A, B**), and details of the PPI were listed in **Table S2**. In addition to the four pathways in **Figure 4**, KEGG pathway enrichment analysis of PPI results revealed that pathways associated with nodule formation included Pathways in cancer and Viral carcinogenesis; pathways associated with viral infection included Human T-cell leukemia virus 1 infection, Human papillomavirus infection, Epstein-Barr virus infection, Kaposi sarcoma-associated herpesvirus infection, and Human cytomegalovirus infection; pathways associated with multiple cellular processes including p53 signaling pathway were also enriched (**Figure 5A**). The four pathways analyzed by GSEA (**Figure 4**) were subjected to KEGG pathway relationship analysis (**Figure 5C**), which indicated that the four pathways were associated with nodule formation included Gastric cancer, Hepatocellular carcinoma, Proteoglycans in cancer, Breast cancer, Endometrial cancer, and Colorectal cancer, pathways associated with multiple cellular processes included MAPK signaling pathway and TGF-beta signaling pathway in addition to the pathways analyzed in **Figure 5A**.

### 3.6 GSEA analysis of differential gene expression profiles

**Figure 6:**
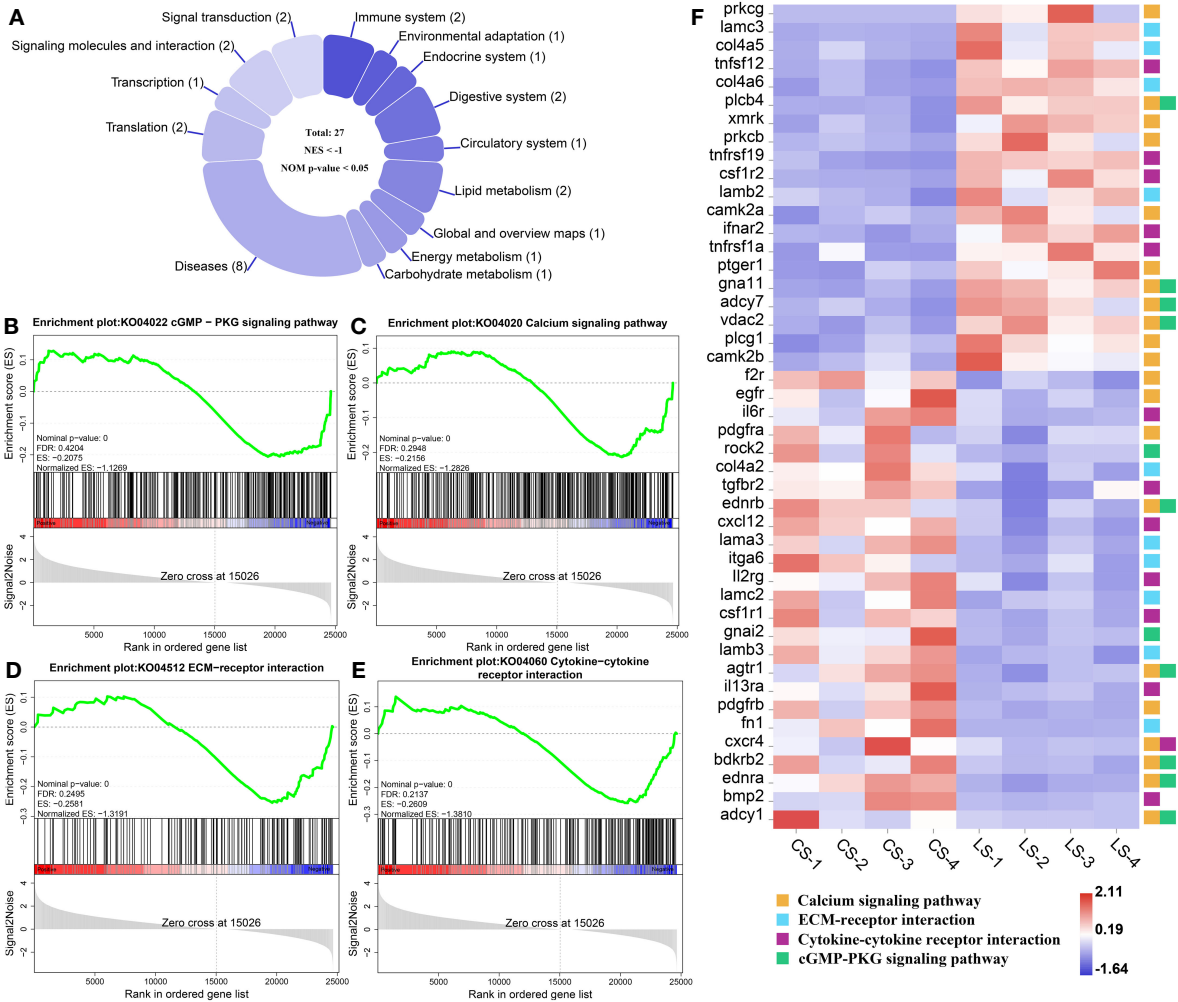
GSEA analysis of down-regulated pathways and related genes associated with nodule formation after LCDV infection of flounder. **(A)** The number of down-regulated pathways. **(B-E)** KEGG pathways associated with nodule formation including cGMP-PKG signaling pathway **(B)**, Calcium signaling pathway **(C)**, ECM-receptor interaction **(D)**, and Cytokine-cytokine receptor interaction **(E)**. **(F)** The heatmap of DEGs. GSEA was used to analyze the signaling pathways enrichment in different groups. Normalized enrichment score (NES) indicated the analysis results across gene sets. Nominal p-value presented if a set was significantly enriched.

In total, there were 27 down-regulated pathways (**Figure 6A**), of which the main ones associated with nodule formation included the cGMP-PKG signaling pathway (**Figure 6B**) and Calcium signaling pathway (**Figure 6C**) in Signal transduction; ECM-receptor interaction (**Figure 6D**) and Cytokine-cytokine receptor interaction (**Figure 6E**) in Signaling molecules and interactions. Genes, including *prkcg*, *lamc3*, *col4a5*, *tnfsf12*, *col4a6*, *plcb4*, *xmrk*, *prkcb*, *tnfrsf19*, *csf1r2*, *lamb2*, *camk2a*, *ifnar2*, *tnfrsf1a*, *ptger1*, *gna11*, *adcy7*, *vdac2*, *plcg1*, and *camk2b*, were all significantly upregulated, while *f2r*, *egfr*, *il6r*, *pdgfra*, *rock2*, *col4a2*, *tgfbr2*, *ednrb*, *cxcl12*, *lama3*, *itga6*, *ll2rg*, *lamc2*, *csf1r1*, *gnai2*, *lamb3*, *agtr1*, *il13ra*, *pdgfrb*, *fn1*, *cxcr4*, *bdkrb2*, *ednra*, *bmp2*, and *adcy1*, were all significantly downregulated (FDR < 0.05, FC ≥ 2) (**Figure 6F**). Details of these genes are listed in **Table S1**.

### 3.7 Visualization of pathways in cancer

**Figure 7:**
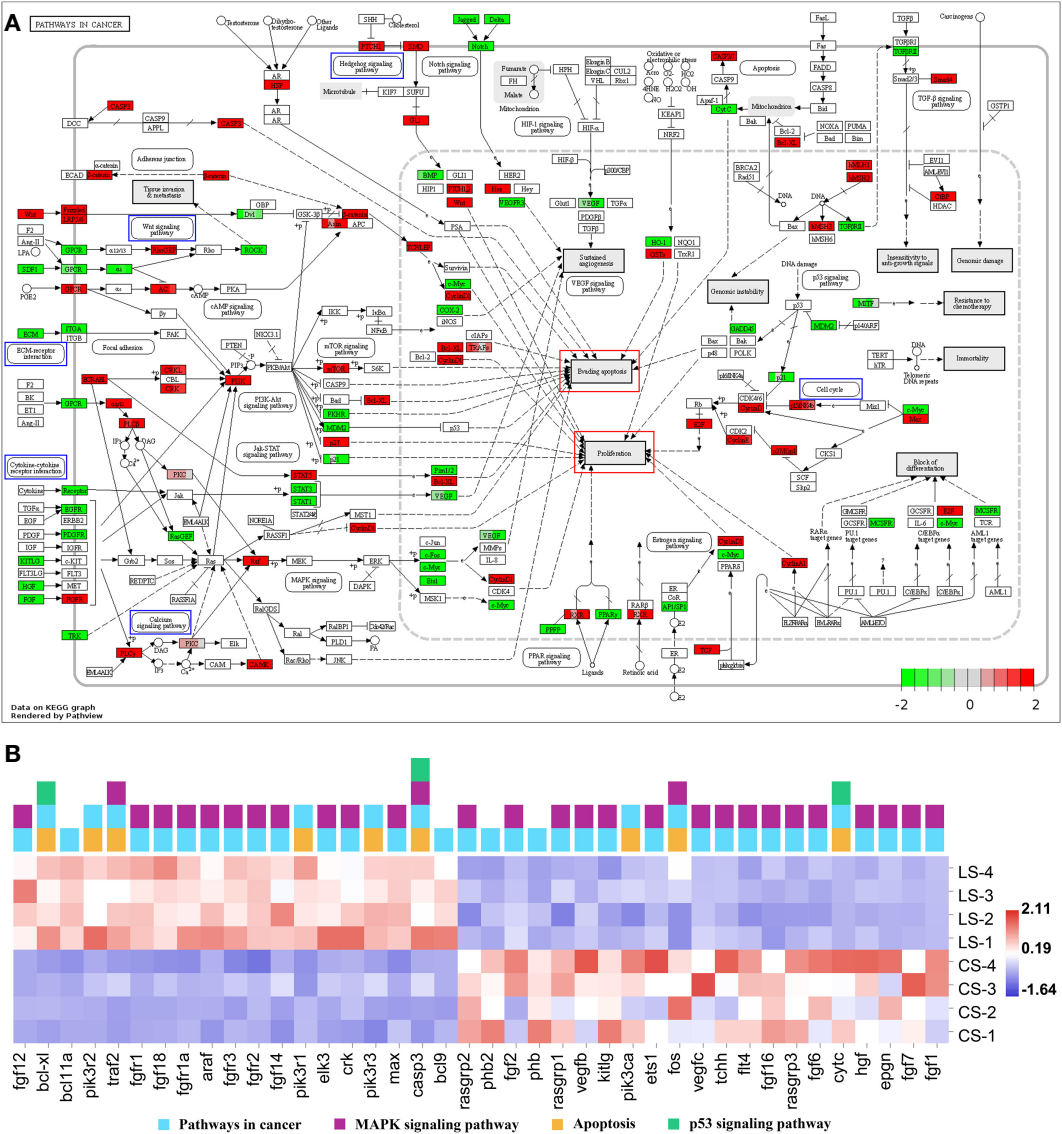
Pathways in cancer from KEGG. **(A)** Visualization of DEGs expression. Blue boxes represented pathways analyzed in **Figures 4B–E** and **6B–E**; red boxes represented cellular effects; DEGs were filled in with color, with red representing up-regulation, green representing down-regulation and shades representing expression size. **(B)** The heatmap of DEGs.

Enrichment of DEGs in **Figures 4F** and **6F** into Pathways in cancer showed that these genes were involved in different signaling pathways, ultimately leading to cellular processes including Evading apoptosis and Proliferation, further suggesting that the pathways analyzed in **Figures 4** and **6** synergistically promote nodule formation (**Figure 7A**).

In addition, the DEGs in Pathways in cancer were visualized and the heat map was analyzed. Among them, except for the DEGs analyzed in **Figures 4** and **6**, *fgf12*, *bcl-xl*, *bcl11a*, *pik3r2*, *traf2*, *fgfr1*, *fgf18*, *fgfr1a*, *araf*, *fgfr3*, *fgfr2*, *fgf14*, *pik3r1*, *elk3*, *crk*, *pik3r3*, *max*, *casp3*, and *bcl9* were significantly upregulated (FDR < 0.05, FC ≥ 2). In contrast, *rasgrp2*, *phb2*, *fgf2*, *phb*, *rasgrp1*, *vegfb*, *kitlg*, *pik3ca*, *ets1*, *fos*, *vegfc*, *tchh*, *flt4*, *fgf16*, *rasgrp3*, *fgf6*, *cytc*, *hgf*, *epgn*, *fgf7*, and *fgf1* were significantly downregulated (FDR < 0.05, FC ≥ 2) (**Figure 7B**).

### 3.8 Tissue expression patterns of focused genes

**Figure 8:**
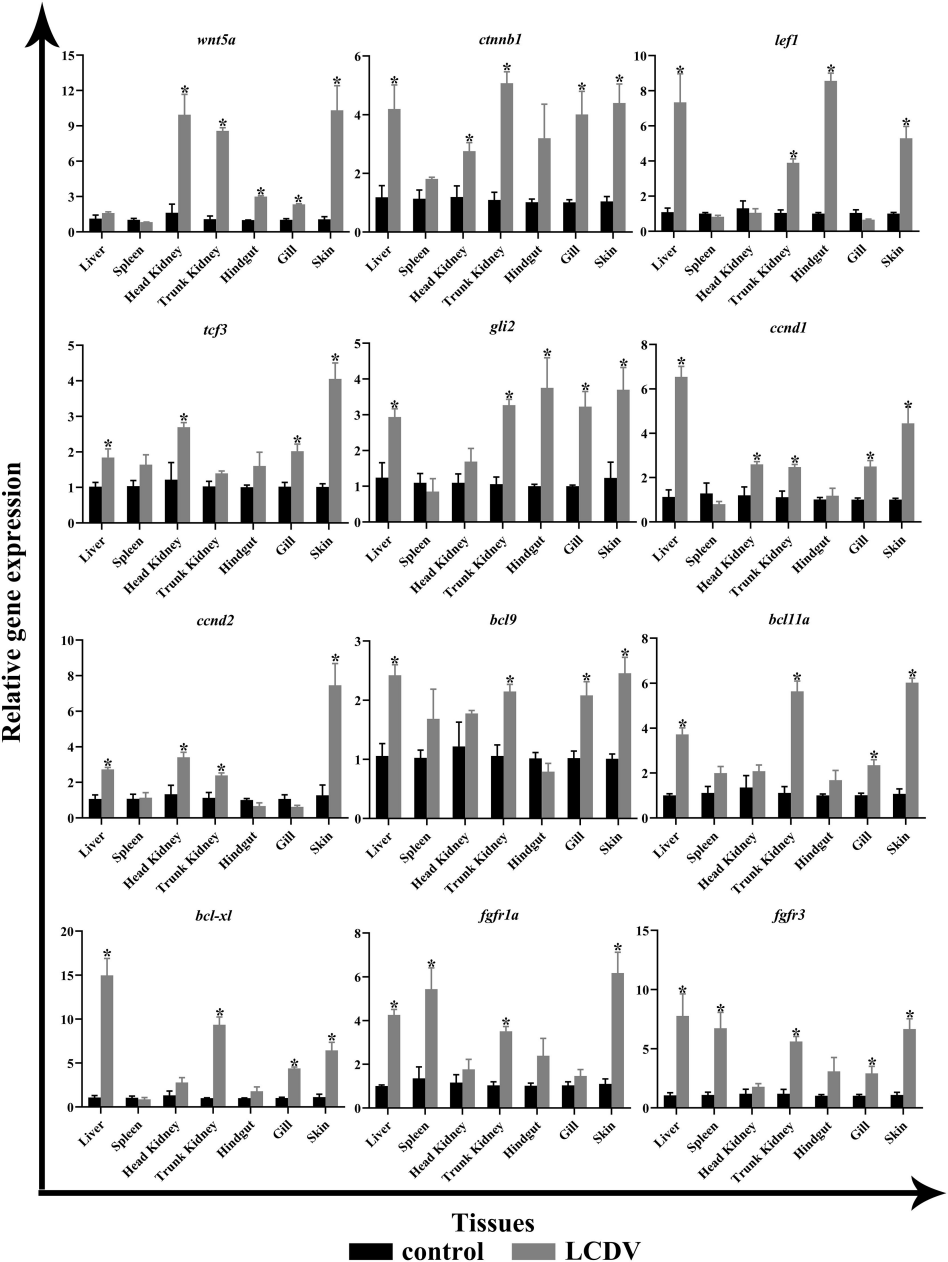
qRT-PCR of key genes of the focal pathway in various tissues of LCDV-infected flounder. The results were presented as the means ± SEM of four individuals. Asterisk indicated significant difference (*p* < 0.05).

The tissue expression patterns of several aforementioned key genes were examined by qRT-PCR, including ligand *wnt5a*, cell cycle-associated *ccnd1* and *ccnd2*, transcription factors *ctnnb1*, *lef1*, *tcf3*, and *gli2*, B-cell lymphoma/leukemia (BCL) family members *bcl9*, *bcl11a*, and *bcl-xl*, cytokine receptors *fgfr1a* and *fgfr3*. In comparison to the LCDV-uninfected group, *wnt5a* expression levels were considerably higher in the head kidney, trunk kidney, hindgut, gills, and skin (*p* < 0.05). The cell cycle-related *ccnd1* and *ccnd2* all had significantly higher levels in the liver, head kidney, trunk kidney, and skin, and *ccnd1* was also significantly overexpressed in the gills (*p* < 0.05). All transcription factors were significantly more highly expressed in liver and skin (*p* < 0.05). In addition, *ctnnb1* and *tcf3* in the head kidney, *ctnnb1*, *lef1*, and *gli2* in the trunk kidney, *lef1* and *gli2* in the hindgut, and *ctnnb1*, *tcf3*, *gli2* in the gills were strongly expressed (*p* < 0.05). BCL family members *bcl9*, *bcl11a*, and *bcl-xl* shared a similar expression pattern that all considerably overexpressed in liver, trunk kidney, gills, and skin (*p* < 0.05). The cytokine receptors *fgfr1a* and *fgfr3* were all significantly highly expressed in the liver, spleen, trunk kidney, and skin, while *fgfr3* was also significantly overexpressed in the gills (*p* < 0.05) (**Figure 8**).

## 4 Discussion

The main target tissues for LCDV include the skin and fin where lymphocystis nodules usually develop. In recent searches, LCDV has been detected in internal organs such as brain, liver, kidney, spleen, and gut in addition to fins and skin (31–33). According to several genetic studies, LCDV primarily affects the skin, gut, liver, and kidney (15, 34–36). In gilthead seabream (*Sparus aurata*), the LCDV genome has been detected in the caudal fin, gut, liver, spleen, kidney, and brain, with the highest viral loads in the caudal fin, followed by the kidney and brain (36). In *Amphiprion ocellaris*, LCDV is detected in the fin and spleen, while in Senegalese sole (*Solea senegalensis*), LCDV MCP gene transcripts are detected in the liver, kidney, brain, gut, and skin/fin at 5-7 dpi (8, 34). Previously, we found that the target tissues of LCDV were skin, liver, spleen, kidney, gills, stomach, heart, and gut in flounder (37). In this study, obvious skin and fin nodules developed in flounder for about one month, and typical hypertrophic cells were present in skin nodules and LCDV particles were observed in the cytoplasm of these cells, while LCDV copy number detection showed the highest viral load in the skin, next in the gills, trunk kidney, hindgut, spleen and liver, and the lowest in the head kidney. Combining our previous study that showed flounder immunized with formalin-inactivated LCDV produced the most powerful immune responses at 21°C in the spleen and head kidney (38), we suggested that the naturally infected flounder in this study had a long course of disease, strong immune responses in the spleen and head kidney might limit viral replication, whereas high viral loads remained in tissues with particularly pronounced foci, especially in the skin nodules. Moreover, studies on Senegalese sole (*Solea senegalensis*) and turbot (*Scophthalmus maximus*) have suggested that LCDV can spread to various tissues by infecting peripheral blood cells (34, 39), and our study has found that LCDV can infect peripheral blood IgM^+^ B cells that support viral replication through a 27.8kDa receptor-mediated mechanism (15), these results provide a rationalization for the detection of LCDV in all tissues of the flounder in this study.

The mechanism of lymphocystis cell formation in fish is less well studied. To date, significant alterations in genes related to cell cycle regulation have been found in flounder with lymphocystis cell formation by using microarray experiments, suggesting that LCDV infection leads to cell cycle arrest (5). In human prostate cancer cells, PHB and PHB2 have tumor suppressor functions, and they interact with the E2F transcription factor family in the nucleus to reduce E2F function, causing cell cycle arrest in the G1/S phase (40, 41). In this study, transcriptome sequencing analysis of skin nodule tissue indicated that the cell cycle was activated with an up-regulation trend of cell cycle protein-related genes, differing from the significant down-regulation of *phb* and *phb2*, which might lead to rapid cell passage through the cycle checkpoint in favor of viral proliferation. Cell cycle is regulated by the upstream pathways, i.e., Wnt signaling pathway and Hedgehog signaling pathway, in which Wnt signaling pathway regulates various physiological processes such as growth control, stem cell renewal, embryonic development, and tissue differentiation (42). β-catenin (*ctnnb1*), the core transcription factor in Wnt signaling pathway, is normally located on the surface of the cell membrane, mostly involved in homotypic cell adhesion, and to a lesser extent in the cytoplasm, but it cannot enter the nucleus. Nevertheless, when mutations in β-catenin occur, β-catenin can accumulate in the cytoplasm and enter the nucleus where it binds to LEF/TCF to cause transcription of target genes, including the cell growth cycle-related genes *ccnd1* and *ccnd2*, causing pathological changes in cell growth, including tumor formation (43). In mammals, several viruses regulate β-catenin through their proteins to promote cell proliferation and tumorigenesis. As oncogenic viruses, KSHV using the LANA protein and EBV using LMP2A activate and stabilize β-catenin, allowing β-catenin to aggregate into the nucleus to regulate the upregulated expression of target genes including *ccnd1* and *myc*, ultimately leading to cell proliferation and even tumorigenesis (44, 45). Similarly, HBV encodes HBx and hepatitis B surface antigen (HBsAg) proteins that silence antagonists of Wnt/β-catenin signaling pathway or upregulate and stabilize its key components, such as β-catenin, causing aberrant transcription of target genes, which drive cell proliferation and ultimately hepatocarcinogenesis (46). PHB can influence the role of WNT family members in cancer. For instance, overexpression of *phb* in human prostate cancer cells decreases the expression of several members of WNT family and reduces the motility and invasiveness of cancer cells, and *phb* plays an important role in the inter-regulation of *wnt7b*, *wnt9a*, and *wnt10b* with the cell cycle (40). In the present study, we found that Wnt signaling pathway was activated and the gene levels of *ctnnb1*, *lef1*, *tcf3*, *ccnd1*, and *ccnd2* were significantly upregulated, while *ctnnb1* was significantly co-expressed with *ccnd1* and *ccnd2* as hub genes in LCDV-infected skin, gills, head kidney, trunk kidney, and liver tissues. Wnt signaling pathway was also found to interact with multiple tumor pathways. These results suggested that LCDV activated Wnt signaling pathway as well as promoted cell proliferation and nodule formation by regulating *ctnnb1* which in turn leads to overexpression of cell cycle proteins. In addition, Wnt signaling pathway is reported to play an important role in the replication of several viruses. Activation of this pathway can promote avian leukosis virus subgroup J (ALV-J) gene expression and virus production in chicken embryonic fibroblasts cells, while inhibition of this pathway limits virus production in chicken embryonic fibroblasts cells and chicken hepatoma cells (47). Wnt/β-catenin signaling pathway is also thought to act in concert with the Bovine herpesvirus type1 latent gene product to maintain latent Bovine herpesvirus type1 infection, with β-catenin playing a central role (48). In this study, we also found that activated Wnt signaling pathway was associated with multiple viral infection pathways, including Human papillomavirus infection and Kaposi sarcoma-associated herpesvirus infection. These findings provided new insights into LCDV virus-host interaction and offered some potential antiviral strategies to control LCDV infection. However, the exact mechanism of which will require later in-depth studies.

The hedgehog signaling pathway was also activated in this study, and based on the KEGG database, we found that there was not only a reciprocal relationship between it and Wnt signaling pathway, but also a link to the tumor formation pathways. In this pathway, when the protein hedgehog (HH) ligand binds to the protein patched homolog (PTCH), smoothened homolog (SMO) repression is removed, and zinc-finger (ZF) transcription factor GLI activity is enhanced, which then enters the nucleus and activates transcription of genes that control cell proliferation, survival and differentiation (49, 50). In this study, *gli2* and *gli3*, members of the GLI family, were significantly upregulated, and they could control cell cycle progression, regulate gene expression levels of cell cycle proteins including *ccnd1* and *ccne2*, and even promote tumourigenesis. It has been reported that silencing of *gli2* leads to cell cycle arrest in G0/G1 phase in human vascular smooth muscle cells and myofibroblasts (51, 52). Similar studies in osteosarcoma, cervical cancer, hepatocellular carcinoma, and hepatocellular carcinogenesis have been conducted to control cell cycle progression (53–56). In cervical cancer, *gli2* overexpression is found to promote cell proliferation, while knockdown of *gli2* causes a stalling effect in G0/G1 phase and a reduction in *ccnd1* gene expression and upregulation of *p21* and *p27* levels (54). In hepatocellular carcinoma, the knockdown of *gli2* gene results in G1 phase arrest, accompanied by downregulation of *ccnd1* and *ccne2* gene expression and upregulation of *p21* levels (55). Additionally, the oncogenic virus HBV is confirmed to contribute to hepatocellular carcinogenesis by regulating members of the GLI family, mainly due to the ability of HBx proteins to stabilize and activate the transcriptional activity of *gli1* and *gli2* (56). In osteosarcoma studies, silencing of *gli2* is found to cause upregulation of *p21*, inhibition of cyclin D1, SKP2, and phosphorylated Rb, thus inducing G1 phase arrest and ultimately preventing the growth of osteosarcoma (53). Basal cell carcinoma is one of the most common types of skin cancer, and Wnt signaling pathway and Hedgehog signaling pathway have been reported to play important roles in this cancer formation (57). Similarly, the present study analyzed a trend of up-regulation in Basal cell carcinoma, which shared the same DEGs with Wnt signaling pathway and Hedgehog signaling pathway, reinforcing the importance of the two pathways in lymphocystis nodule formation. Considering that *gli2* was expressed in a consistent pattern with *ccnd1* and *ccnd2* in skin, gill, trunk kidney, and liver tissues infected by LCDV in our study, we speculated that the Hedgehog signaling pathway might act in concert with Wnt signaling pathway to control the cell cycle and cause the formation of lymphocystis in fish.

Inhibition of apoptosis is thought to be a major factor in lymphocystis cell formation. By using microarray assay, the reference has investigated gene expression changes in the fins of LCDV-infected flounders and concluded that lymphocystis cell formation was mainly due to inhibition of apoptosis, including the down-regulation of caspase-3 precursor (*casp3*), caspase-6 precursor (*casp6*), caspase-8 precursor (*casp8*) and many other apoptosis-inducing genes (5). Unlike these results, *casp3* was found to be significantly up-regulated in the present study, while some members of the BCL family associated with cell proliferation and inhibition of apoptosis, including *bcl9*, *bcl11a*, and *bcl-xl*, were also considerably up-regulated in LCDV-infected skin, gill, trunk kidney, and liver tissues. BCL9/BCL9L binding to β-catenin can significantly affect tumor growth, suggesting that BCL9/BCL9L interacting with β-catenin plays a key role in tumor progression (58). Bcl11 gene family includes *bcl11a* and *bcl11b*, of which *bcl11a* is a proto-oncogene. In patients with Hodgkin lymphoma, *bcl11a* expression is found to be elevated and associated with EBV infection (59). Bcl-xl is an important member of the BCL-2 family and plays a crucial role in the inhibition of apoptosis (60). A study on HIV shows that β-catenin protects HIV-infected lymphocytes from apoptosis by directly activating the *bcl-xl* promoter activity to induce its expression (61). Therefore, up-regulation of *bcl9*, *bcl11a*, *bcl-xl*, and aforementioned *ctnnb1* in this study revealed that LCDV might regulate the BCL family through β-catenin to promote cell proliferation and inhibit apoptosis. Nevertheless, previous transcriptome analysis of flounder gills infected with LCDV indicates that genes associated with apoptosis including TNF ligand superfamily member 13B and TNF receptor-1 were up-regulated (6). Similarly, the present study found significant upregulation of *tnfsf12*, *tnfrsf1a*, and *tnfrsf19*, these genes might exert a pro-apoptotic effect which appeared to be detrimental to lymphocystis cell formation. But some studies have also found that LCDV can create cytoplasmic TNF receptor-like proteins after *in vivo* infection which react with multiple apoptotic or proliferative signaling proteins, thus inhibiting the apoptotic cascade downstream of the TNFR superfamily (62). In addition, the transcriptome results from flounder gills also demonstrate a downregulation of the apoptosis inhibitor *bcl-2* (6), which shows an opposite expression pattern to the BCL family members that inhibit apoptosis in this study. Combining these results, we suggested that skin cells infected by LCDV might initiate apoptosis to prevent the spread of the virus, while LCDV relied on its own proteins to interfere with apoptosis to facilitate proliferation, which was more like a competition between the virus and the host cells. VDAC2 was confirmed in our previous study to act as a functional receptor mediating the entry of LCDV into flounder gill (FG) cells (12). In the present study, *vdac2* was significantly upregulated, and we believe that it contributes to lymphocystis formation mainly because it also plays an important role in endogenous apoptosis. VDAC is responsible for the release of apoptosis-inducing proteins such as cytochrome C from the mitochondria into the cytoplasm to induce apoptosis, a process that is influenced by the competitive interactions of pro-apoptotic and anti-apoptotic factors with VDAC isoforms (63, 64). The anti-apoptotic factors Bcl-2, Bcl-xL, or hexokinases block the binding of VDAC to pro-apoptotic proteins by interacting with VDAC to close the pore and thus prevent the release of cytochrome C (63). Additionally, at the gene level, we found that *cytc* was significantly down-regulated, suggesting that cytochrome C was inhibited at both the protein and gene levels thereby failing to exert its pro-apoptotic role. References have shown that effective pro-apoptotic factor bax-mediated apoptosis is dependent on vdac2 (65), whereas the anti-apoptotic factor bcl-xl exerts an anti-apoptotic effect by blocking bax damage to the outer mitochondrial membrane (66). Moreover, LCDV produces the TNF receptor analog and VDAC can be oligomerized by TNF-α, which may contribute to the inhibition of apoptosis by LCDV (3). VDAC can interact with viral proteins to reduce apoptosis in infected cells, as shown in Infectious Bursal Disease Virus whose VP5 formed a complex with RACK1 and VDAC2 to inhibit apoptosis (67). RACK1 is also a receptor for LCDV entry into FG cells together with VDAC2, and not only that, the gene expression levels of RACK1 reach a peak later than VDAC2 after LCDV infection (12). In this study, RACK1 showed an up-regulation trend although it was not significant (**Table S1**), this might due to the difference in the expression time of the two receptors. Therefore, RACK1 as a receptor for LCDV entry might also play a role in lymphocystis formation. However, whether LCDV proteins forming a complex with VDAC2/RACK1 can inhibit host cell apoptosis, and whether VDAC2/RACK1 determines the balance between cell death and survival (that is, apoptosis and lymphocystis cell formation), need to be further investigated.

Fibroblast growth factors (FGFs) bind to their receptors, fibroblast growth factor receptors (FGFRs), and activate the downstream signaling pathways they regulate, playing an important role in both pro-mitotic (embryogenesis, growth, and development) and non-mitotic (neuromodulation, metabolic regulation) biological processes. Among these pathways, high expression and mutations of fgfr lead to abnormal activation of the signaling pathway, resulting in uncontrolled pro-division and subsequent tumor production (68), for example, significant expression of *fgfr2* has been detected in cervical cancer (69). In addition, it is shown that the addition of the pan FGFR inhibitor AZD4547 alone inhibits the growth of cells associated with E2/E4/E5 in HPV positive tumor (70). In the present study, the FGFR family members, *fgfr1*, *fgfr2*, and *fgfr3*, were significantly upregulated in the skin nodule tissue of LCDV-infected flounder. *Fgfr3* is the first member of the FGFR family, which undergoes somatic mutations in tumors and is expressed at elevated levels in cell lines through repeated translocations to and from immunoglobulin heavy chain (IGH) sites (71, 72). *Fgfr1* is thought to be similar to *fgfr3* in recurrent translocations in some tumors (73), but little functional validation has been reported except that it promotes tumorigenesis through gene amplification and overexpression (74). FGFRs also play a role in viral infection, and fgfr1 acts as a co-receptor for adeno-associated virus type 3 (AAV-3) strain H (75). So, it is reasonable to infer that FGFRs might play a similar role in lymphocystis formation after LCDV infection, but more evidence is required to clarify this.

The P53 signaling pathway is involved in several cellular processes and is particularly important in suppressing tumor formation. Several oncogenic viruses promote tumor formation by modulating the p53 signaling pathway. For example, HBV inhibits p53-induced apoptosis by suppressing p53 activity through its HBx protein (17, 76). HCV affects the DNA-binding function of p53 through its NS5A protein (17). While HPV E6/E7 proteins and MCPyV tumor antigen inhibit and degrade p53 and affect other pathways closely associated with cancer including the Notch signaling pathway and TGF-beta signaling pathway (77–80). In addition, oncogenic viruses often manipulate the MAPK signaling pathway to promote host cell proliferation and cause cell metastasis. EBV activates the MAPK signaling pathway through the LMP1 protein to contribute to nasopharyngeal carcinoma cell invasion (81). KSHV activates the MK2 kinase, an effector of the MAPK signaling pathway, through the kaposin B protein to promote tumor formation (82). In this study, although there was no significant trend of up- and down-regulation of the p53 signaling pathway, TGF-beta signaling pathway, and MAPK signaling pathway, we found that there was a linkage of these pathways with Wnt signaling pathway and Cell cycle, implying that there were common DEGs of these pathways that promoted the formation of lymphocystis cells.

In summary, we performed RNA-seq on skin nodule tissues of naturally LCDV-infected flounder using high-throughput sequencing technology and analyzed transcriptome data, and found that Wnt signaling pathway, Hedgehog signaling pathway, Cell cycle, and Basal cell carcinoma associated with lymphocystis formation were activated. These pathways regulated cell cycle-related genes such as *ccnd1* and *ccnd2* through key genes such as *ctnnb1* and *gli2* to promote cell proliferation, which has been shown to interact with several viral infection and tumor formation pathways, and therefore they are considered to be an important cause of lymphocystis formation. *Bcl9*, *bcl11a*, and *bcl-xl*, members of the BCL family that promote cell proliferation and inhibit apoptosis, were significantly upregulated, as were *fgfr1*, *fgfr2*, and *fgfr3*, which are closely related to tumor formation. *Tnfsf12*, *tnfrsf1a*, and *tnfrsf19*, which are associated with pro-apoptosis, as well as *vdac2*, which promotes viral replication by inhibiting apoptosis, also acts as a receptor for LCDV entry, were significantly upregulated. Pro-apoptotic *cytc* and anti-tumor *phb* and *phb2* were significantly down-regulated. These results are expected to explain the molecular basis of lymphocystis formation after LCDV infection of flounder. For the first time, we have analyzed the pathways and differentially expressed genes associated with lymphocystis formation using high-throughput sequencing technology, providing a new breakthrough in the study of lymphocystis formation in fish.

## Data availability statement

The datasets presented in this study can be found in online repositories. The names of the repository/repositories and accession number(s) can be found below: https://www.ncbi.nlm.nih.gov/, SAMN36345915; https://www.ncbi.nlm.nih.gov/, SAMN36345916; https://www.ncbi.nlm.nih.gov/, SAMN36345917; https://www.ncbi.nlm.nih.gov/, SAMN36345918; https://www.ncbi.nlm.nih.gov/, SAMN36345919; https://www.ncbi.nlm.nih.gov/, SAMN36345920; https://www.ncbi.nlm.nih.gov/, SAMN36345921; https://www.ncbi.nlm.nih.gov/, SAMN36345922.

## Ethics statement

The animal study was approved by the Animal Care and Use Committee of Ocean University of China (permit number: OUC-AE-2022-071). The study was conducted in accordance with the local legislation and institutional requirements.

## Author contributions

HZ: Data curation, Formal Analysis, Investigation, Writing – original draft, Methodology, Validation. XS: Conceptualization, Formal Analysis, Funding acquisition, Writing – original draft, Writing – review & editing, Project administration. XT: Formal Analysis, Resources, Writing – review & editing, Methodology, Validation. JX: Formal Analysis, Writing – review & editing, Resources, Validation. HC: Formal Analysis, Resources, Writing – review & editing, Validation. WZ: Conceptualization, Funding acquisition, Writing – review & editing, Supervision.
